# ﻿A new toad of *Oreolalax* Myers & Leviton, 1962 (Anura, Megophryidae) from Sichuan Province, southwest China

**DOI:** 10.3897/zookeys.1212.122222

**Published:** 2024-09-16

**Authors:** Yin Meng Hou, Pu Yang Zheng, Hao Qi Yu, Bin Wang, Xiao Hong Chen, Feng Xie

**Affiliations:** 1 College of Life Sciences, Henan Normal University, Xinxiang 453007, China Chengdu Institute of Biology, Chinese Academy of Sciences Chengdu China; 2 Department of Herpetology, Chengdu Institute of Biology, Chinese Academy of Sciences, Chengdu 610041, China Henan Normal University Xinxiang China; 3 The Observation and Research Field Station of Taihang Mountain Forest Ecosystems of Henan Province, Xinxiang 453007, China The Observation and Research Field Station of Taihang Mountain Forest Ecosystems of Henan Province Xinxiang China; 4 University of Chinese Academy of Sciences, Beijing 100049, China University of Chinese Academy of Sciences Beijing China; 5 College of Life Sciences, Sichuan University, Chengdu 610065, Sichuan, China Sichuan University Chengdu China

**Keywords:** 16S, COI, Hengduan Mountains, new species, *Oreolalax* species, taxonomy

## Abstract

A new species of the genus *Oreolalax* Myers & Leviton, 1962 is described from Sichuan Province, southwest China. Molecular phylogenetic analyses based on mitochondrial gene sequences clustered the new species as an independent clade nested with *O.rugosus*, *O.liangbeiensis*, and *O.major*. The new species could be distinguished from its congeners by a combination of the following characters: body size moderate (39.8–52.8 mm in male); head broad; tympanum absent; interorbital region with dark triangular pattern; 1/3 toes webbed, with broad lateral fringes, belly smooth, brown yellow or medium yellow scattered variable brown spots; skin on dorsum relatively rough with fine tiny and large warts granules; middle pectoral glands are evident in males; flanks with dark-brown warts granules; upper surface of limbs with dark bars; and iris orange above and creamy-white below. The new species inhabits subtropical alpine scrub and swamp.

## ﻿Introduction

*Oreolalax* Myers & Leviton (1962) belongs to the family MegophryidaeBonaparte (1850) (Amphibian, Anura), LeptobrachiinaeDubois (1980). In 1962, Myers and Leviton established the genus using *Oreolalaxpingii* as the type species, including four species, *Oreolalaxpingii* Liu, 1943, *Oreolalaxpopei* Liu, 1947, *Oreolalaxrugosus* Liu, 1943 and *Oreolalaxschmidti* Liu, 1947. With the deepening of morphological, ecological and molecular studies, the taxonomic status of the genus has been gradually established (Tian and Jiang 1986; Fei et al. 1989, 1990; Dubois and Ohler 1998; Fu et al. 2007; Pyron and Wiens 2011).

Based on morphological differences, molecular divergence, and phylogenetic placement, *Oreolalaxsterlingae* Nguyen, Phung, Le, Ziegler & Böhme, 2013 from northeast Vietnam and *Oreolalaxlongmenmontis* Hou, Shi, Hu, Deng, Jiang, Xie & Wang, 2020 from eastern Hengduan Mountains have been described, showing controversial internal cladistic relationships within the genus (Nguyen et al. 2013; Hou et al. 2020). As of now, 19 species have been recorded in this genus, distributed in southwest China and the northernmost part of Vietnam (Frost 2024), inhabiting mountain streams at 700–3300 m a.s.l. (Wei et al. 2009; Fei et al. 2012).

Hengduan Mountains, located in the southeastern part of Qinghai-Tibet Plateau, have a complex terrain and significant vertical climate changes. The mountain ecosystem in this region boasts a diverse range of habitats, capturing global attention for its biodiversity, and its speciation and protection have been of concern. During the field surveys in 2023 in Yanyuan City, Sichuan Province (Prov.), southeastern Hengduan Mountains, China, we collected eight adult and seven tadpole *Oreolalax* specimens. Our detailed morphological comparisons and molecular phylogenetic analyses indicated that these specimens should represent an undescribed species. Herein we describe it as a new species.

## ﻿Materials and methods

### ﻿Sample

**Figure 1.**
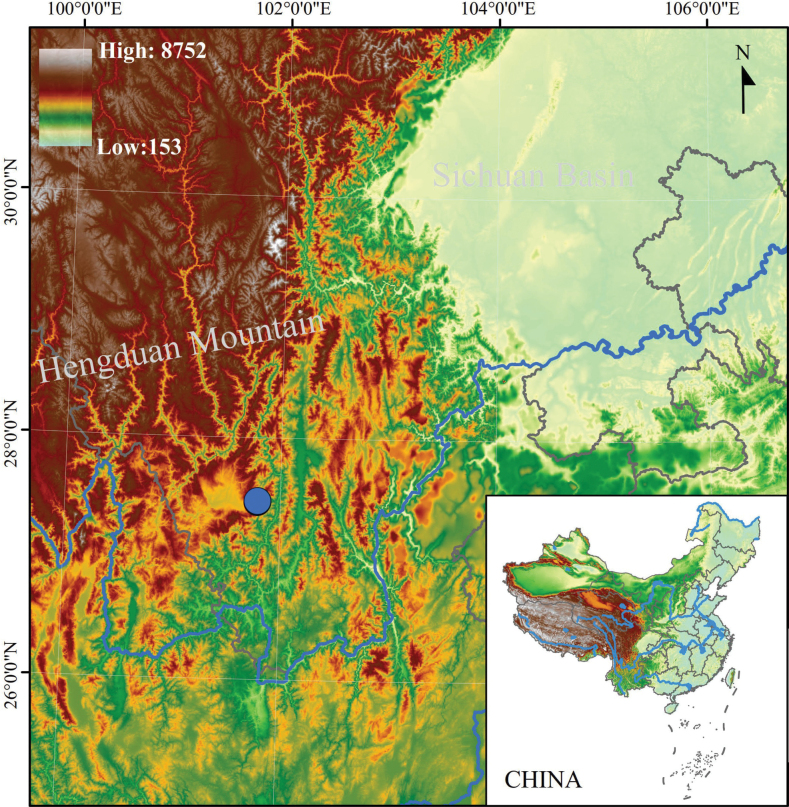
Location of the type locality of *Oreolalaxyanyuanensis* sp. nov., Shuhe town, Sichuan Province, China.

Through a field survey in June 2023, a total of 15 samples of the undescribed species including eight adult males and seven tadpoles were collected nocturnally from Shuhe town, Yanyuan County, Sichuan Province, China (Suppl. material 1; Fig. 1). Taxonomic assignments of tadpoles were confirmed by molecular results. After taking photographs, the toads and tadpoles were euthanized using isoflurane, and the specimens were fixed and preserved in 75% ethanol. Tissue samples were taken and preserved separately in 95% ethanol prior to fixation. Specimens were deposited in Chengdu Institute of Biology, Chinese Academy of Sciences (**CIB**, **CAS**).

### ﻿Molecular phylogenetic analyses

Genomic DNA from each specimen collected in this work was extracted using a TIANamp Genomic DNA Kit by TIANGEN (BEIJING) BIOTECH, China. Fragments of the mitochondrial genes 16S rRNA and cytochrome *c* oxidase I (COI) genes were amplified. Primer sequences were retrieved from the literature for 16S (Simon et al. 1994) and COI (Che et al. 2012). PCR amplifications for the 16S/COI gene were performed in a 25 mL volume reaction with the following conditions: an initial denaturing step at 95 °C for 4 min; 36 cycles of denaturing at 95 °C for 40 s, annealing at 55 °C/50 °C for 40 s and extending at 72 °C for 70 s, and a final extending step of 72 °C for 10 min. Sequencing was conducted using an ABI3730 automated DNA sequencer at Sangon Biotechnologies Co., Ltd. (Shanghai, China).

For phylogenetic comparisons, *Scutigerboulengeri* Bedriaga, 1898 was selected as an outgroup. Sequences of 16 *Oreolalax* species and outgroups were downloaded from GenBank, and seven of *Oreolalax* species (*O.rugosus*, *O.pingii*, *Oreolalaxmajor* Liu & Hu, 1960, *O.schmidti*, *Oreolalaxliangbeiensis* Liu, Hu & Fei, 1979, *Oreolalaxgranulosus* Fei, Ye & Chen, 1990, and *O.popei*) were uploaded to GenBank in this study (Accession numbers: PP272909–PP272951, Table 1).

**Table 1.** Information for samples used in molecular phylogenetic analyses in this study. “–” represents a lack of data.

Species	Voucher	Locality	GenBank accession number	
			16S	CO1
*Oreolalaxyanyuanensis* sp. nov.	CIBSH20230603kd01	Yanyuan, Liangshan, Sichuan, China	PP272915	PP272937
*O.yanyuanensis*	CIBSH20230603kd02	Yanyuan, Liangshan, Sichuan, China	PP272916	PP272938
*O.yanyuanensis*	CIBSH20230603kd03	Yanyuan, Liangshan, Sichuan, China	PP272917	PP272939
*O.yanyuanensis*	CIBSH20230603kd04	Yanyuan, Liangshan, Sichuan, China	PP272918	PP272940
*O.yanyuanensis*	CIBSH20230603kd05	Yanyuan, Liangshan, Sichuan, China	PP272919	PP272941
*O.yanyuanensis*	CIBSH20230603kd06	Yanyuan, Liangshan, Sichuan, China	PP272920	PP272942
*O.yanyuanensis*	CIBSH20230603kd07	Yanyuan, Liangshan, Sichuan, China	PP272921	PP272943
*O.yanyuanensis*	CIBSH20230603016	Yanyuan, Liangshan, Sichuan, China	PP272922	PP272944
*O.yanyuanensis*	CIBSH20230603017	Yanyuan, Liangshan, Sichuan, China	PP272923	PP272945
*O.yanyuanensis*	CIBSH20230603018	Yanyuan, Liangshan, Sichuan, China	PP272924	PP272946
*O.yanyuanensis*	CIBSH20230603019	Yanyuan, Liangshan, Sichuan, China	PP272925	PP272947
*O.yanyuanensis*	CIBSH20230603020	Yanyuan, Liangshan, Sichuan, China	PP272926	PP272948
*O.yanyuanensis*	CIBSH20230603021	Yanyuan, Liangshan, Sichuan, China	PP272927	PP272949
*O.yanyuanensis*	CIBSH20230603023	Yanyuan, Liangshan, Sichuan, China	PP272928	PP272950
*O.yanyuanensis*	CIBSH20230603024	Yanyuan, Liangshan, Sichuan, China	PP272929	PP272951
*O.multipunctatus*	CIB2013wb091	Emei, Sichuan, China	NC_037382	NC_037382
*O.xiangchengensis*	CIB20130642	Xiangcheng, Sichuan, China	MH727696	MH727696
*O.lichuanensis*	–	Hubei, China	KU096847	KU096847
*O.rhodostigmatus*	–	Suiyang, Guizhou, China	MF770485	MF770485
*O.jingdongensis*	–	Xujiaba, Jingdong, Yunnan, China	MF953479	MF953479
*O.omeimontis*	CIBEMS18061205	Emei, Sichuan, China	MN688660	OP247647
*O.nanjiangensis*	CIBSCNJNJ2006004	Shibatan, Nanjiang, Sichuan, China	MN688658	–
*O.sterlingae*	IEBR A.2012.1	Sa Pa, Lao Cai, Vietnam	KC569981	–
*O.longmenmontis*	CIB20180526001	Pengzhou, Sichuan, China	MN688670	OP247644
*O.rugosus*	CIBSCJFGYC201301	Zhaojue, Liangshan, Sichuan, China	PP272909	PP272930
*O.pingii*	CIBSC20130521004	Zhaojue, Liangshan, Sichuan, China	PP272910	PP272931
*O.major*	CIBEM1824	Emei, Sichuan, China	MN688655	PP272932
*O.schmidti*	CIBEM1820	Emei, Sichuan, China	PP272911	PP272933
*O.liangbeiensis*	WG20180538	Puxiong, Yuexi, Sichuan, China	PP272912	PP272934
*O.granulosus*	CIBYN20130305023	Ailoushan, Jingdong, Yunnan, China	PP272913	PP272935
*O.popei*	CIB2020061508	Baoxing, Ya’an, Sichuan, China	PP272914	PP272936
*Scutigerboulengeri*	GGS-PBX2-16	Kangding, Sichuan, China	OK584750	OK544538

Sequences were assembled and aligned using Mega v. 7.0 (Kumar et al. 2016) A total of 1084 bp (16S+COI gene) sequences were obtained from the *Oreolalax* species. The maximum likelihood method (**ML**) and Bayesian inference (**BI**) method were used to construct gene trees to analyze the intraspecific phylogenetic relationship of species using PhyloSuite v. 1.2.2 (Zhang et al. 2020). The best substitution models were inferred by the corrected Akaike information criterion (**AICc**) using the ModelFinder module in PhyloSuite software (BI: (GTR+I+G model for all partition); IQ: (GTR+F+R2: 16S, TPM2u+F+I+G4: COI)); in the ML analyses, we performed 1000 ultrafast bootstrap replicates based on the IQ-TREE. In the BI analyses, two runs each with four Markov chains were run for 20 million iterations with sampling every 1000 generations. The first 25% of generations were removed at the “burn-in” stage followed by calculation of Bayesian posterior probabilities and the 50% majority-rule consensus of the post burn-in trees sampled at stationarity. Trees were visualized with the FigTree v. 1.4.2 program (Rambaut 2016). Finally, genetic distance was calculated with the pairwise uncorrected *p*-distance model between *Oreolalax* species on the COI gene using MEGA v. 7 (Kumar et al. 2016).

### ﻿Morphological analyses

Measurements were made with a digital caliper to the nearest 0.1 mm. The terminology and methods followed Fei et al. (2009) and Watters et al. (2016). Twenty-three morphometric characters were measured for adults: **SVL** (snout–vent length), direct line distance from tip of snout to posterior margin of vent; **HW** (head width), at the widest point of the jaws angle; **IOD** (interorbital distance), the shortest distance between the anterior corners of the orbits; **HL** (head length), from the posterior of the jaws to the tip of the snout; **ED** (eye diameter), horizontally from the anterior to posterior corner of the eye; **SL** (snout length), distance from the tip of the snout to the anterior corner of the eye; **UEW** (upper eyelid width), greatest width of the upper eyelid margins, measured perpendicular to the anterior-posterior axis; **IND** (internarial distance), shortest distance between the inner margins of the nostrils; **EN** (eye–nostril distance), from anterior corner of the eye to the posterior margin of the nostril; **NS** (snout–nostril length), distance from the center of the external nares to the tip of the snout; **LAHL** (length of lower arm and hand), the length from the elbow to the end of the third finger; **FAW** (forearm width), greatest width of the forearm; **THL** (thigh length), distance from the vent to the knee; **TL** (tibia length), distance from the outer surface of the flexed knee to the heel/tibiotarsal inflection; **TW** (tibia width), maximum width of tibia along its length; **FL** (foot length), from the base of the inner metatarsal tubercle to the tip of Toe IV; **LFT** (length of foot and tarsus), the length from the tibial appendicular joint to the end of the fourth toe; **Toe4L** (toe IV length), from the metatarsal tubercle to the tip of Toe IV; **Fin1L** (finger I length), from the proximal edge of the palmar tubercle to the tip of the Finger I; **Fin3L** (finger III length), from the proximal edge of the palmar tubercle to the tip of the Finger III; **IMT** (inner metatarsal tubercle length), the greatest length of the inner metatarsal tubercle; **IPTL** (inner palmar tubercle length), maximum length of the inner palmar tubercle; **OPTL** (outer palmar tubercle length), maximum length of the outer palmar tubercle, measured parallel along forearm axis.

Thirteen morphometric characters were measured for tadpoles: **BH** (maximum body height); **BW** (maximum body width); **SVL** (snout–vent length); **MW** (mouth width), distance between two corners of mouth; **SL** (snout length), distance from the tip of the snout to the anterior corner of the eye; **SS** (snout to spiraculum), distance from spiraculum to the tip of the snout; **ED** (maximum eye diameter); **IND** (internasal distance),minimum distance between nostrils; **IOD** (interocular distance), minimum distance between eyes; **TAH** (tail height), maximum height between upper and lower edges of tail; **TAL** (tail length), distance from base of vent to the tip of tail; **TBW** (maximum width of tail base); **TOL** (total length), distance from the tip of the snout to the tip of tail.

## ﻿Results

### ﻿Molecular phylogenetic analyses

**Figure 2.**
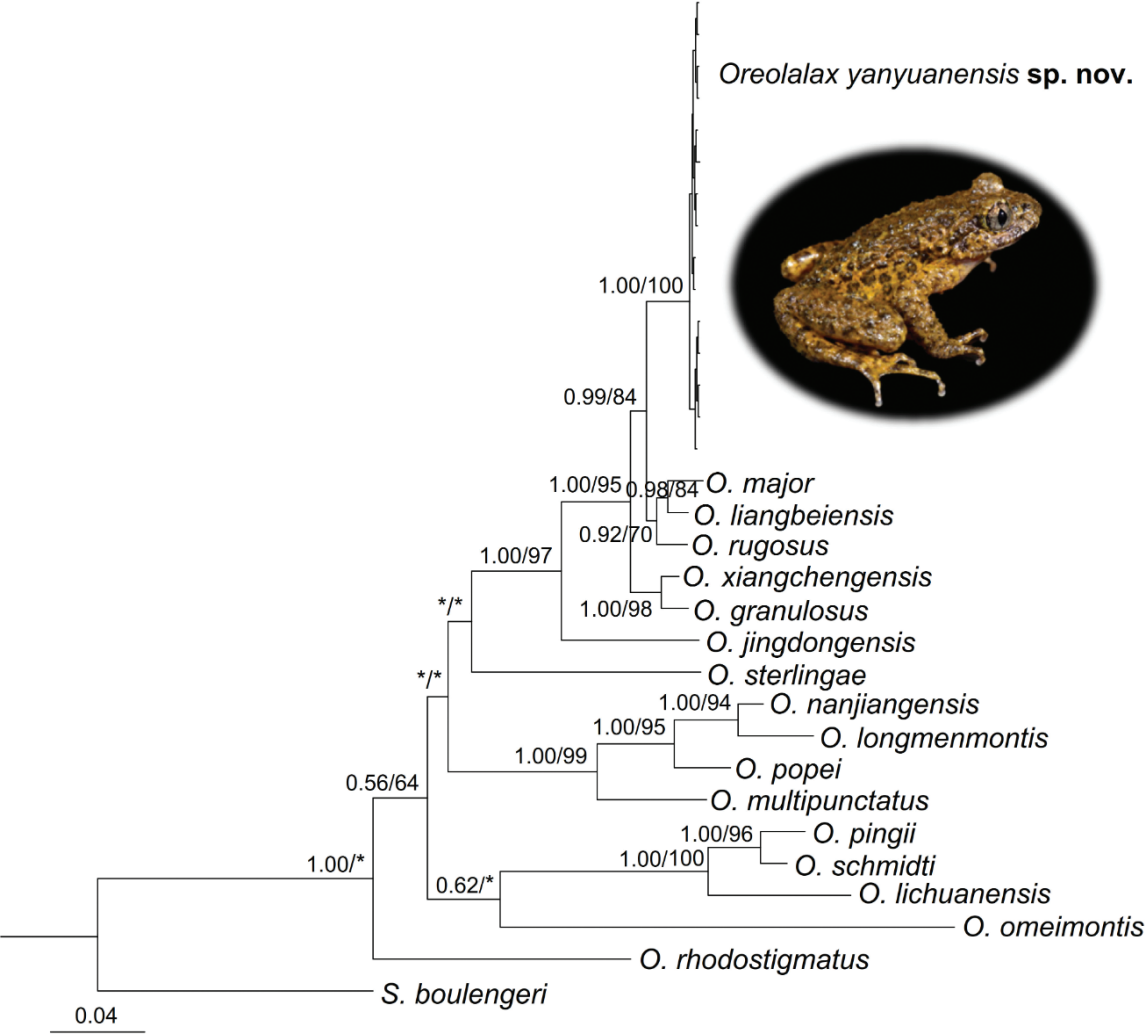
Bayesian inference (BI) tree of *Oreolalax* species based on the mitochondrial 16S and COI gene sequences. ML bootstrap support/Bayesian posterior probability is denoted beside node. The “*” represents posterior probability ≤0.5/ bootstrap value ≤50.

Phylogenetic results based on 16S and COI genes showed that the topological structures obtained by BI and ML analyses resulted in essentially identical topologies (Fig. 2). All samples of the undescribed species occupied an independent monophyly and were closely related to *O.rugosus*, *O.major*, and *O.liangbeiensis*. Genetic distances on the *CO*I gene between all samples of the undescribed species were 0.0%–0.3%. The undescribed species is closest to *O.rugosus* on genetic distance (4.5%), being higher than, or at the same level, as many pairs of substantial species, such as *O.liangbeiensis* vs *O.major* (3.8%), *O.liangbeiensis* vs *O.rugosus* (3.3%) (Suppl. material 2).

### ﻿Morphological analyses

We also compared morphological characters of the undescribed species with other *Oreolalax* species. Comparative morphological data were obtained from the literature for *Oreolalaxchuanbeiensis* Tian, 1983 (Tian 1983), *O.granulosus* (Fei et al. 1990), *Oreolalaxjingdongensis* Ma, Yang & Li, 1983 (Yang et al. 1983), *O.liangbeiensis* (Liu et al. 1979), *Oreolalaxlichuanensis* Liu, Hu & Fei, 1979 (Liu et al. 1979), *O.major* (Liu and Hu 1960), *Oreolalaxmultipunctatus* Wu, Zhao, Inger & Shaffer, 1993 (Wu et al. 1993), *Oreolalaxnanjiangensis* Fei & Ye, 1999 (Fei et al. 1999), *Oreolalaxomeimontis* Liu & Hu, 1960 (Liu and Hu 1960), *O.pingii* (Liu 1943), *O.popei* (Liu 1947), *Oreolalaxpuxiongensis* Liu, Hu & Fei, 1979 (Liu et al. 1979), *Oreolalaxrhodostigmatus* Liu, Hu & Fei, 1979 (Liu et al. 1979), *O.rugosus* (Liu 1943), *O.schmidti* (Liu 1947), *O.sterlingae* (Nguyen et al. 2013), *Oreolalaxweigoldi* Vogt, 1924 (Vogt 1924), and *Oreolalaxxiangchengensis* Fei & Huang, 1983 (Fei and Huang 1983), and *O.longmenmontis* (Hou et al. 2020). Specimens were examined for comparison: 16 *Oreolalax* specimens (holotype, paratype, and topotype) from Chengdu Institute of Biology, Chinese Academy of Sciences (CIB, CAS) (Suppl. material 3).

#### ﻿ Oreolalax yanyuanensis sp. nov.

Taxon classificationAnimaliaAnuraMegophryidae

52D41E83-3A41-504F-8801-4FBD39AA1316

https://zoobank.org/D770076A-DB34-4045-AEF9-FFFBC2FBA5D8

Figs 3 , 4 , 5 , 6

##### Type material.

**Figure 3.**
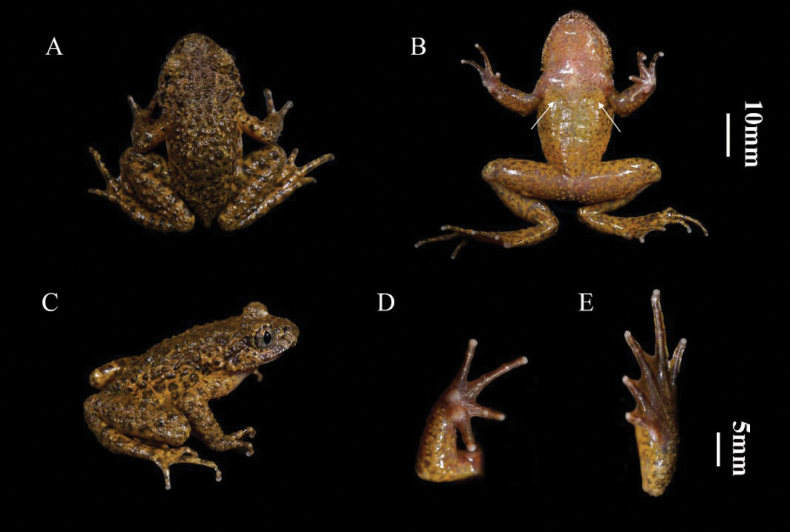
Photos of the holotype CIBSH20230603020 of *Oreolalaxyanyuanensis* sp. nov. **A** dorsal view **B** ventral view; the white arrow indicates the position of the pectoral gland **C** lateral view **D** ventral view of hand **E** ventral view of foot.

***Holotype*.** • CIBSH20230603020 (Fig. 3), adult male, collected by F. Xie from Shuhe town, Yanyuan county, Sichuan Province (27.473443°N, 101.789624°E, 3108 m a.s.l.) China. ***Paratypes*.** • Seven adult males collected from a small stream of Shuhe town (27.475205°N, 101.789108°E, 3127 m a.s.l.); on June 3, 2023 (CIBSH20230603016–19, CIBSH20230603021, CIBSH20230603023–24) by P.Y. Zheng, H.Q. Yu and F. Xie.

##### Other specimens.

• Seven tadpoles (CIBSH20230603kd01–07) collected from a small stream of Shuhe town (27.466241°N, 101.786933°E, 3032 m a.s.l.); on June 3, 2023; by P.Y. Zheng, H.Q. Yu and F. Xie.

##### Diagnosis.

The new species is assigned to genus *Oreolalax* based on following characters: maxillary teeth prominent; back rough, scattered with large warts, covered with oval black spots; pupil vertical; tongue moderately broad, notched behind; femoral glands prominent; pectoral and axillary gland present in males.

The new species differs from its congeners by a combination of the following characters: body size moderate 39.8–52.8 mm in male; head broad; tympanum hidden; distinct black spots present on flanks; toes 1/3 webbed, with broad lateral fringes; dorsal body deep brown or yellowish-brown; belly smooth, middle yellow, scattered fine mottling, abdominal margin more spotted; skin on dorsum rough with dense and varied size granules, warts are covered with dark spots or no spots; iris light orange or light yellow above, creamy silver white bellow; and middle pectoral glands are evident in males.

##### Holotype description

**(Fig. 3).** Body size moderate (SVL 47.4 mm). Head width greater than length (HW 17.6 mm, HL 16.2 mm); maxillary teeth developed, without vomerine teeth or acoustic sac; snout bluntly rounded in dorsal view, slightly projecting over lower jaw, longer than eye diameter (SL 6.3 mm, ED 5.9 mm); canthus rostralis indistinct, interorbital distance (IOD 5.0 mm) wider than internarial distance (IND 3.5 mm), distinctly larger than upper eyelid (UEW 3.0 mm); nostrils oval, closer to tip of snout than eyes (EN 3.1 mm, NS 3.0 mm); no tympanic membrane; supratympanic fold broad; tongue moderately broad, notched behind; pupil vertical.

Fingers moderate, relative finger lengths: I < II < IV < III; finger tips slightly dilated; subarticular tubercles absent; inner palmar tubercle large, nearly rounded, outer palmer tubercle small, oval, completely separated.

Hindlimbs relatively long, length 177% of body length; shank length subequal to thigh length, slightly shorter than foot length (THL 23.4 mm, TL 23.8 mm, FL 24.4 mm); heels partially overlap when thighs are positioned at right angles to the body and tibia-tarsal articulation reaches the middle eye when leg stretched; toes 1/3 webbed with distinct fringes; inner metatarsal tubercle long oval, small.

In life, dorsal body and head rough, back with moderate sparse granules, relatively small warts on head; with dark-brown triangular between eyes; dorsal arms and hindlimbs with small granules and bumps; distinct warts cover the fold and posterior of snout. Ventral skin smooth; pectoral glands flat; pectoral glands evident, chest spines and finger spines not visible; femoral glands slightly swollen, distinct on posterior thigh. Small verrucous granules around the anus.

Large brown markings on dorsum, dark brown triangular pattern between eyes; large markings dorsum brown; ventral skin medium yellow, with scattered little dark speckling; supratympanic fold dark brown; lateral head and flanks brown with dark patches; throat mixed pink and orange yellow, margin with small beige warts; chest pink and the pectoral glands medium yellow; forelimbs covered with black irregular spots; dorsal limbs yellowish-brown, the spots and stripes of dorsal upper arms and tibiotarsal articulation black; ventral arms, thigh, tibia medium yellow with flesh marking; upper iris light orange yellow, lower iris creamy white, both parts embedded black mesh lines.

**Figure 4.**
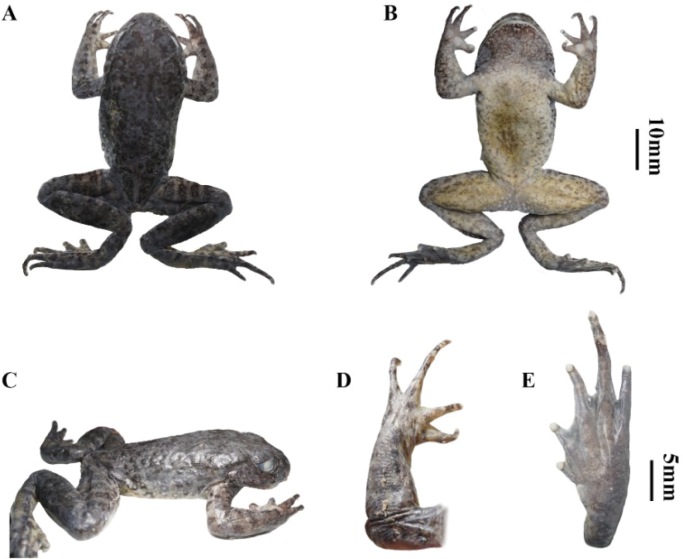
The holotype CIBSH20230603020 of *Oreolalaxyanyuanensis* sp. nov. in preservative **A** dorsal view **B** ventral view **C** lateral view **D** dorsal view of the left hand **E** ventral view of the left foot.

In preservative (75% ethanol), dorsal body and head dark grey; irregular spots in forelimbs, black longitudinal stripes on hindlimbs; ventral surface beige, throat and arms beige white; with grayish-brown speckling; mandibular margin warts white; pectoral glands and ventral of the hindlimb beige yellow, scattered black spots; hand and feet dark grey, finger tips and palms grayish-white, inner metatarsal tubercle grey; lateral grey on snout and undereye, patches black; skins beneath supratympanic fold dark grey, flanks grey, covered with creamy white warts, black spots around the edges of warts. Perianal warts and femoral gland creamy-white (Fig. 4).

##### Tadpoles.

**Figure 5.**
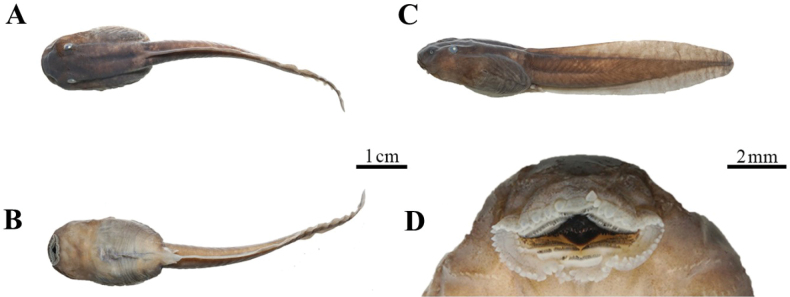
Photos for the tadpole CIBSH20230603kd01 in preservative of *Oreolalaxyanyuanensis* sp. nov. **A** dorsal view **B** ventral view **C** lateral view **D** ventral view of head.

Measurements see Suppl. material 4. Description based on preserved tadpole CIBSH20230603kd01 at Gosner stage 37 (TOL 62.3 mm, BL 20.6 mm) (Fig. 5). The mouth is located below the rostral end; labial tooth row formula I:(5+5) /(5+5):I; the upper lip papillae is large, the central missing length is close to 3 papillae position, the lower lip papillae is small and pointed; and there are small teeth on the auxiliary processes of the oral corner; jaw sheaths strong, serrated, the lip teeth are daggerlike; body elliptical in dorsal view, body width is 113% of height; snout rounded, eye positioned dorsolateral; SL 29% of BL; eyes relatively small, ED 8.3% of BL; nostrils near oval; tail long and muscled, TAL 210% of BL; TAH 113% of BH; TBW 44% of BW; SS 53% of BL. Body dark brown in the back and lateral view, creamy yellow in the ventral; tail brown; the single opening of the spiracle lateral, without a free distal tube; tail end blunt; faint brown cloud spots faintly visible on upper caudal fin, caudal fin light and broad.

##### Variation.

Measurements variation of specimens see Suppl. material 1 (unit in mm). Half of the individuals had faint markings on the abdomen (CIBSH20230603016, CIBSH20230603018, CIBSH20230603021, CIBSH20230603023), and half had more markings on the abdomen (CIBSH20230603017, CIBSH20230603019, CIBSH20230603020, CIBSH20230603022). Color of each specimen was brownish-yellow (CIBSH20230603018, CIBSH20230603023), medium brown (CIBSH20230603016, CIBSH20230603019, CIBSH20230603021, CIBSH20230603022), or dark brown (CIBSH20230603017, CIBSH20230603020). Dorsal markings are obvious in most individuals (CIBSH20230603016–22), except one (CIBSH20230603023). Lateral markings are obvious in most individuals (CIBSH20230603016–21, CIBSH20230603023), except one (CIBSH20230603022). Color of warts covering the back was yellowish-brown (CIBSH20230603022), light brown (CIBSH20230603023), brown (CIBSH20230603016, CIBSH20230603020), dark brown (CIBSH20230603018, CIBSH20230603019), black (CIBSH20230603017, CIBSH20230603021). Wart size was relatively small in some individuals (CIBSH20230603017, CIBSH20230603020, CIBSH20230603021, CIBSH20230603023) and relatively large in others (CIBSH20230603016, CIBSH20230603018, CIBSH20230603022, CIBSH20230603023). Half of the specimens had dark temporal folds (CIBSH20230603017, CIBSH20230603018, CIBSH20230603020, CIBSH20230603021), in others the folds were generally lighter (CIBSH20230603016, CIBSH20230603019, CIBSH20230603022, CIBSH20230603023).

Measurements variation of tadpoles see Suppl. material 4. The dorsal color of CIBSH20230606kd01, CIBSH20230606kd03–04, CIBSH20230606kd05–06 is nearly black, while numbers CIBSH20230606kd02 and CIBSH20230606kd07 are dark brown. Light brown cloud spots on the upper caudal fin, ranged from faintly visible (CIBSH20230606kd01, CIBSH20230606kd03) to obvious (CIBSH20230606kd02, CIBSH20230606kd04–07).

##### Comparisons.

In *Oreolalax*, 19 species occur in southwest China and northern Vietnam. *Oreolalaxyanyuanensis* sp. nov. could be easily distinguished from them by several characters (Suppl. material 5). By having moderate body size (39.8–52.8 mm) in males, the new species differs from *O.major* (vs. 59.2–68.7 mm), *O.popei* (vs. 60.0–69.0 mm), *O.rhodostigmatus* (vs. 57.5–73.5 mm), *O.sterlingae* (vs. 36.8 mm), and *O.weigoldi* (vs. 58.2 mm).

By having head width > head length, the new species differs from *O.chuanbeiensis*, *O.nanjiangensis* (vs. head width ≈ head length), *O.weigoldi* (vs. head width = head length), *O.multipunctatus*, *O.popei*, *O.rhodostigmatus*, and *O.schmidti* (vs. head width < head length).

By having no tympanum, the new species differs from *O.liangbeiensis*, *O.major*, *O.longmenmontis*, *O.sterlingae*, *O.chuanbeiensis*, *O.multipunctatus*, *O.nanjiangensis*, *O.pingii*, *O.popei*, *O.puxiongensis*, *O.schmidti*, *O.weigoldi* (vs. hidden), *O.lichuanensis*, *O.omeimontis* (vs. concealed or slightly visible), and *O.rhodostigmatus* (vs. rather visible).

By having 1/3 toe webbing, the new species differs from *O.puxiongensis*, *O.schmidti* (vs. no webbing), *O.longmenmontis*, *O.sterlingae*, *O.lichuanensis*, *O.multipunctatus*, *O.nanjiangensis*, *O.omeimontis*, *O.pingii*, *O.popei*, *O.rhodostigmatus* (vs. rudimentary), *O.xiangchengensis*, and *O.weigoldi* (vs. well webbed).

By having triangular pattern between eyes, the new species differs from *O.rugosus*, *O.liangbeiensis*, *O.major*, *O.xiangchengensis*, *O.sterlingae*, *O.chuanbeiensis*, *O.granulosus*, *O.lichuanensis*, *O.nanjiangensis*, *O.pingii*, *O.popei*, *O.rhodostigmatus*, and *O.weigoldi* (vs. no triangular pattern).

By having middle spiny patches on the chest, the new species differs from *O.liangbeiensis*, *O.major*, *O.xiangchengensis*, *O.chuanbeiensis*, *O.granulosus*, *O.weigoldi*, *O.omeimontis* (vs. large patches), *O.pingii*, *O.rhodostigmatus*, O.jingdongensis, *O.lichuanensis* (vs. relatively large patches), *O.longmenmontis*, *O.sterlingae*, *O.multipunctatus*, *O.nanjiangensis*, and *O.popei* (vs. small patches).

By having dark bars on the limbs, the new species can differ from *O.rugosus* (vs. no or irregular), *O.xiangchengensis*, *O.pingii*, and *O.puxiongensis* (vs. no).

By having brown yellow or medium yellow scattered variable brown spots on the belly, the new species can differ from *O.rugosus* (creamy yellow or yellow, no spots), *O.liangbeiensis* (creamy white without any spots), *O.xiangchengensis* (light brown, no spots), *O.sterlingae* (cream with dark marbling), *O.granulosus* (yellow-white or with fine light gray veins), *O.lichuanensis* (purplish with dark brown flecks), *O.multipunctatus* (grey brown, with few or without spots), *O.nanjiangensis* (without dark spots), *O.pingii* (gray-white, no spots), *O.popei* (brown-red, fully covered with small gray-brown spots), *O.puxiongensis* (grayish-yellow, no spots), *O.rhodostigmatus* (grayish-brown, no spots), *O.schmidti* (entirely purple-yellow, no spots), *O.weigoldi* (light brown with dark cloudy spots on ventrolateral), and *O.longmenmontis* (flesh red and greyish-white with some black speckles).

**Figure 6.**
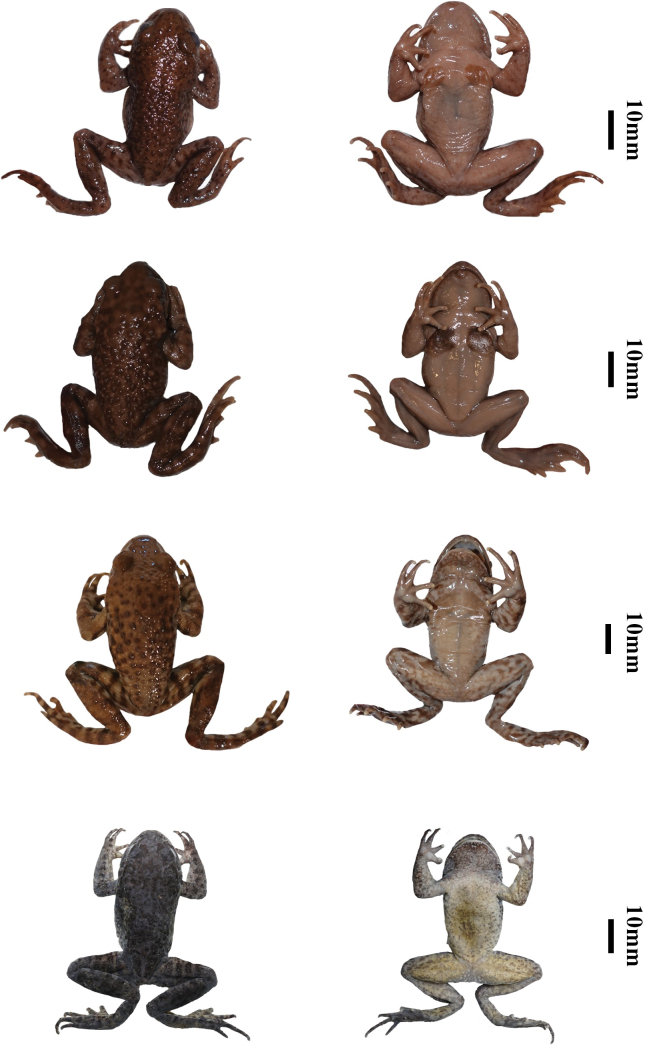
Specimen photos of *Oreolalaxyanyuanensis* sp. nov. and its relative species **A, B** dorsal and ventral view of the holotype CIB25333 of *O.rugosus***C, D** dorsal and ventral view of the topotype CIB24678 of *O.liangbeiensis***E, F** dorsal and ventral view of the topotype CIB24695 of *O.major***G, H** dorsal and ventral view of the topotype CIBSH20230603020 of *Oreolalaxyanyuanensis* sp. nov. Scale bar: equal to 10 mm.

*Oreolalaxyanyuanensis* is genetically closest to *O.rugosus*, *O.liangbeiensis* and *O.major*. In addition to the morphological differences (Fig. 6; Suppl. material 5), the new species distinctly differs from these three in measurement proportions. The new species can differ from *O.rugosus* by having larger LFT, TL, and smaller IN, IOD, UEW, TW. The new species distinctly differs from *O.liangbeiensis* by having larger HL, HW, ED, LAHL, TL, LFT and smaller SL, IOD. The new species distinctly differs from *O.major* by having larger HW, ED, TL, LFT and smaller SL, IN, IOD (Table 2).

**Table 2.** Morphometric comparisons between *Oreolalaxyanyuanensis* sp. nov. and its relatives. Shaded values represent a ratio of body measurements to SVL that differs between these three species and the new species.

Measurements/SVL (%)	*Oreolalaxyanyuanensis* sp. nov.	*O.rugosus*	*O.liangbeiensis*	*O.major*
	8♂♂	10♂♂	20♂♂	6♂♂
HL	35.9 (34.2–37.4)	35.4	33.3	35.4
HW	37.6 (36.6–39.5)	37.5	35.2	35.8
SL	14.3 (13.2–15.3)	14.6	15.5	15.5
IND	9.0 (7.4–10.3)	10.4	9.8	10.4
IOD	10.1 (9.2–10.6)	11.5	10.9	10.8
ED	11.9 (11.5–12.5)	12.2	10.1	10.5
UEW	8.4 (6.3–9.4)	9.9	9.2	9.0
FAW	10.3 (8.9–12.4)	11.5	10.9	10.8
LAHL	54.6 (51.2–58.0)	51.6	51.0	55.0
TL	51.3 (49.6–53.2)	47.8	45.0	48.0
TW	11.3 (9.2–13.6)	13.7	12.3	11.1
LFT	77.5 (74.3–80.2)	67.5	69.0	73.8
FL	50.8 (47.1–54.6)	48.1	48.7	50.9

In elevational distribution, the new species (occurring between 3000–3200 m) can be distinguished from some *Oreolalax* species occurring below 3000 m (a.s.l.) as follows: *O.major* (vs. 1600–2000 m), *O.longmenmontis* (vs. 1300–1450 m), *O.chuanbeiensis* (vs. 2000–2200 m), *O.granulosus* (vs. 2300–2450 m), *O.jingdongensis* (vs. 2300–2450 m), *O.lichuanensis* (vs. 1790–1840 m), *O.multipunctatus* (vs. 1800–1920 m), *O.nanjiangensis* (vs. 1600–1856 m), *O.omeimontis* (vs. 1050–1800 m), *O.popei* (vs. 1000–2000 m), *O.puxiongensis* (vs. 2600–2900 m), *O.rhodostigmatus* (vs. 700–1790 m), *O.schmidti* (vs. 1700–2400 m), and *O.sterlingae* (vs. 2900 m).

##### Etymology.

The specific epithet “yanyuan” refers to the type locality of the species, Yanyuan County, Sichuan Province. We suggested the common name as “Yanyuan toothed toad”, and the Chinese name as “Yan Yuan Chi Chan (盐源齿蟾)”.

##### Distribution and ecology.

**Figure 7.**
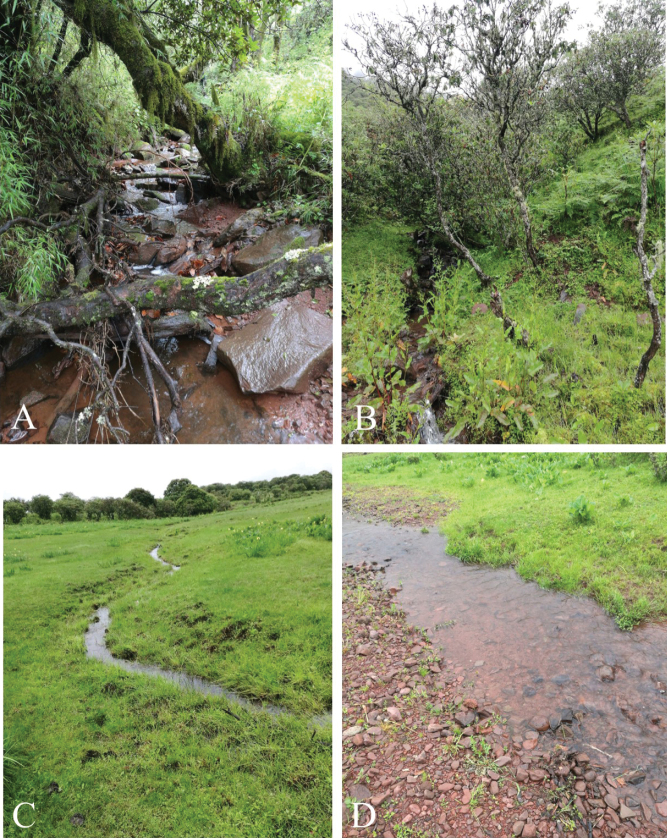
Habitats of *Oreolalaxyanyuanensis* sp. nov. in southeastern Hengduan Mountains region, Sichuan Province, China **A, B, C** adults’ habitats **D** tadpoles’ habitat.

*Oreolalaxyanyuanensis* sp. nov. is currently only known from the type locality, Shuhe town, Yanyuan county, Sichuan Prov., China at elevations of 3000–3200 m. The new species inhabits subtropical alpine scrub and swamp, and was found in small montane streams (Fig. 7). The breeding season is currently uncertain; it is speculated that it breeds in April or May based on the tadpole development stage. Four sympatric amphibian species (*Bombinamaxima* Boulenger, 1905 (Boulenger 1905), *Ranachaochiaoensis* Liu, 1946 (Liu 1946), *Panophrysbinchuanensis* Ye & Fei, 1995 (Ye and Fei 1995) and *Nanoranasichuanensis* Dubois, 1987 (Dubois 1987 “1986”)) were found in the same habitat.

## ﻿Discussion

Hengduan Mountains is part of the hotspot defined as “Mountains of Southwest China”, and its biodiversity conservation has attracted much attention (CEPF 2024). At present, most *Oreolalax* species are distributed in the eastern Hengduan Mountains and the surrounding mountains of the Sichuan Basin at an altitude of 700–3550 m. More than half of *Oreolalax* species is listed as “threatened” by IUCN due to habitat loss, habitat degradation, logging, tourism development, and invasive species (Fei and Ye 2016; Jiang et al. 2016; IUCN 2024). Twelve species of *Oreolalax* are listed as vulnerable, near threatened, endangered, or critically endangered (IUCN 2024). Furthermore, some species have only been recorded at their type locality, and their habitat is not covered by any biodiversity conservation network, even with the high extinction risk (e.g., *O.puxiongensis*). Also, *O.longmenmontis*, which has not yet been evaluated by IUCN, faces low population and habitat loss (Hou et al. 2020). Assessments of habitat status, breeding activity, population size and dynamics are needed for these groups, especially for the newly discovered (e.g., *O.yanyuanensis* sp. nov.) and data deficient species (e.g., *O.weigoldi*).

The auditory system is critical for animals’ survival and reproduction. Studies have shown that the thin air and low air density at high altitudes lead to slow sound speed, and animals singing in anoxic environment at high altitudes will consume a lot of energy, posing a threat to survival (Liao and Liu 2008; Wen 2014). Some amphibians have reduced investment in acoustic communication, resulting in structural degradation of acoustic communication organs which may be adapted to the high-altitude environment (Lehr and Trueb 2007; Wen 2014). In *Oreolalax*, the tympanic membranes are hidden or absent in most groups except *O.rhodostigmatus*. Some high-altitude groups (>3000 m), such as *O.rugosus* and *O.xiangchengensis*, showed the absence of columella, while the low-altitude groups showed a developed columella (Wei et al. 2009). *Oreolalaxyanyuanensis* is distributed at high altitudes (3000–3200 m), but it is not clear whether the ear structure is degraded. Due to the limited number of specimens, we will use Micro-CT scans to explore the middle ear structure in the future.

In June 2023, the individuals of *O.yanyuanensis* found in the wild apparently lacked finger spines and chest spines, and no female individuals have been collected, so it is possible that its breeding season had ended. Based on its morphological characteristics, number and size of tadpoles, it is speculated that *O.yanyuanensis* may reproduce in April or May. Further studies are needed to investigate its reproductive behavior and population dynamics.

## ﻿Conclusions

Based on morphological and molecular evidence, we revealed a new toad belonging to the *Oreolalax* species group—*O.yanyuanensis* sp. nov. The new species is so far only known from Shuhe town, Yanyuan County, south Sichuan Prov., China. The findings in this study improve our understanding of species diversity in the genus *Oreolalax*. More studies are necessary to uncover the population size, reproductive ecology, and habitat status to better protect the new *Oreolalax* species.

## Supplementary Material

XML Treatment for Oreolalax yanyuanensis

## ﻿Additional information

### Author ORCIDs

Yin Meng Hou https://orcid.org/0000-0002-4216-9678

Bin Wang https://orcid.org/0000-0001-6036-5579

Feng Xie https://orcid.org/0000-0001-9532-9586

### Data availability

All of the data that support the findings of this study are available in the main text or Supplementary Information.

## Supplementary materials

Supplementary material 1 Measurements of *Oreolalaxyanyuanensis* sp. nov. Data type xlsx Explanation note Units in mm. See abbreviations for characters in the Materials and methods section. https://binary.pensoft.net/file/1134584This dataset is made available under the Open Database License (http://opendatacommons.org/licenses/odbl/1.0/). The Open Database License (ODbL) is a license agreement intended to allow users to freely share, modify, and use this Dataset while maintaining this same freedom for others, provided that the original source and author(s) are credited.Yin Meng Hou, Pu Yang Zheng, Hao Qi Yu, Bin Wang, Xiao Hong Chen, Feng Xie

Supplementary material 2 Uncorrected *p*-distance between *Oreolalax* species on the COI gene Data type xlsx https://binary.pensoft.net/file/1134585This dataset is made available under the Open Database License (http://opendatacommons.org/licenses/odbl/1.0/). The Open Database License (ODbL) is a license agreement intended to allow users to freely share, modify, and use this Dataset while maintaining this same freedom for others, provided that the original source and author(s) are credited.Yin Meng Hou, Pu Yang Zheng, Hao Qi Yu, Bin Wang, Xiao Hong Chen, Feng Xie

Supplementary material 3 Examined specimens in this study Data type xlsx https://binary.pensoft.net/file/1134586This dataset is made available under the Open Database License (http://opendatacommons.org/licenses/odbl/1.0/). The Open Database License (ODbL) is a license agreement intended to allow users to freely share, modify, and use this Dataset while maintaining this same freedom for others, provided that the original source and author(s) are credited.Yin Meng Hou, Pu Yang Zheng, Hao Qi Yu, Bin Wang, Xiao Hong Chen, Feng Xie

Supplementary material 4 Measurements of tadoples of *Oreolalaxyanyuanensis* sp. nov. Data type xlsx Explanation note Units in mm. See abbreviations for morphometric characters in Materials and methods section. https://binary.pensoft.net/file/1134587This dataset is made available under the Open Database License (http://opendatacommons.org/licenses/odbl/1.0/). The Open Database License (ODbL) is a license agreement intended to allow users to freely share, modify, and use this Dataset while maintaining this same freedom for others, provided that the original source and author(s) are credited.Yin Meng Hou, Pu Yang Zheng, Hao Qi Yu, Bin Wang, Xiao Hong Chen, Feng Xie

Supplementary material 5 Morphological differences between males of *Oreolalaxyanyuanensis* sp. nov. and males of its congeners Data type xlsx https://binary.pensoft.net/file/1134588This dataset is made available under the Open Database License (http://opendatacommons.org/licenses/odbl/1.0/). The Open Database License (ODbL) is a license agreement intended to allow users to freely share, modify, and use this Dataset while maintaining this same freedom for others, provided that the original source and author(s) are credited.Yin Meng Hou, Pu Yang Zheng, Hao Qi Yu, Bin Wang, Xiao Hong Chen, Feng Xie
